# Effects of exercise training on pulmonary vessel muscularization and right ventricular function in an animal model of COPD

**DOI:** 10.1186/s12931-014-0117-y

**Published:** 2014-09-28

**Authors:** Erlend Hassel, Anne Marie Berre, Anne Jarstein Skjulsvik, Sigurd Steinshamn

**Affiliations:** Department of Circulation and Medical Imaging, Norwegian University of Science and Technology, Trondheim, Norway; Department of Pathology, St. Olavs Hospital, Trondheim, Norway; Department of Thoracic Medicine, St. Olavs Hospital, Trondheim, Norway

**Keywords:** COPD, Exercise, Animal model, Cardiac function, Right ventricle, Pulmonary vessel remodeling

## Abstract

**Background:**

Right ventricular dysfunction in COPD is common, even in the absence of pulmonary hypertension. The aim of the present study was to examine the effects of high intensity interval training (HIIT) on right ventricular (RV) function, as well as pulmonary blood vessel remodeling in a mouse model of COPD.

**Methods:**

42 female A/JOlaHsd mice were randomized to exposure to either cigarette smoke or air for 6 hours/day, 5 days/week for 14 weeks. Mice from both groups were further randomized to sedentariness or HIIT for 4 weeks. Cardiac function was evaluated by echocardiography and muscularization of pulmonary vessel walls by immunohistochemistry.

**Results:**

Smoke exposure induced RV systolic dysfunction demonstrated by reduced tricuspid annular plane systolic excursion. HIIT in smoke-exposed mice reversed RV dysfunction. There were no significant effects on the left ventricle of neither smoke exposure nor HIIT. Muscularization of the pulmonary vessels was reduced after exercise intervention, but no significant effects on muscularization were observed from smoke exposure.

**Conclusions:**

RV function was reduced in mice exposed to cigarette smoke. No Increase in pulmonary vessel muscularization was observed in these mice, implying that other mechanisms caused the RV dysfunction. HIIT attenuated the RV dysfunction in the smoke exposed mice. Reduced muscularization of the pulmonary vessels due to HIIT suggests that exercise training not only affects the heart muscle, but also has important effects on the pulmonary vasculature.

**Electronic supplementary material:**

The online version of this article (doi:10.1186/s12931-014-0117-y) contains supplementary material, which is available to authorized users.

## Background

Chronic obstructive pulmonary disease (COPD) is a serious and highly prevalent condition associated with high mortality, morbidity and reduced quality of life. The disease is caused by chronic inflammation due to inhalation of cigarette smoke or other noxious particles which can lead to remodeling of the small airways or destruction of the lung parenchyma and emphysema. COPD is primarily a disease of the lungs and airways and it is defined by expiratory airflow obstruction, however, the disease also has several extrapulmonary manifestations and comorbidities, e.g. cardiovascular disease, osteoporosis, cachexia and skeletal muscle wasting [1].

One of the important complications of COPD is RV failure, which is associated with worse outcomes and increased mortality [2,3]. The development of RV failure is thought to be linked to increased afterload due to pulmonary hypertension (PH), but the PH associated with COPD is usually mild compared to PH of other causes [4]. A recent study has demonstrated that RV dysfunction is common, even in COPD patients with only slightly increased pulmonary artery pressure [5]. This indicates either that the increase in pulmonary artery pressure needed to affect the RV is minimal, or that also other mechanisms than PH are in effect.

Exercise training has been shown to improve exercise endurance, dyspnea, functional capacity and quality of life for COPD patients [6]. The mechanisms through which these improvements are achieved are only partially understood. There are some evidence suggesting that peripheral muscle adaptations play a significant role leading to improved work economy, which again leads to reduced ventilatory requirement for exercise at a given intensity [7,8]. Endurance training improves several risk factors for cardiovascular disease in COPD, including blood pressure and pulse wave velocity, which might indicate improved cardiovascular function [9]. One study has shown that endurance training increases the maximum oxygen uptake and improves both left and right systolic ventricular function in COPD patients without hypoxemia [10]. It is not known by which mechanisms the improvement in cardiac function is achieved, that is whether the function of the heart muscle *per se* is improved (i.e. contractility), or there is an improvement in the conditions under which the heart works (i.e. preload, afterload).

The aim of the present study was to assess the cardiac function in an animal model of COPD, with focus on the right side of the heart. Further we wanted to investigate the potential of exercise training to correct the induced heart dysfunction. We used a mouse model of cigarette smoke induced emphysema. Our model had high levels of carbon monoxide, which is shown to lead to right ventricular hypertrophy [11], indicating right ventricular strain. In the present study the cardiorespiratory fitness, hemodynamic effects and pulmonary artery remodeling were examined to find adaptations to cigarette smoke exposure and subsequent exercise intervention with special emphasis on the pulmonary circulation and the right side of the heart.

## Methods

### Animals and exposure

42 female A/JOlaHsd mice (9 weeks old at start of exposure) were obtained from Harlan Laboratories UK and acclimatized in our laboratory for at least two weeks. The animals were randomized to either cigarette smoke exposure (CS) or fresh air sham exposure (FA), and further to either interval training (IT) or being sedentary (Se), giving four different groups: 1. Fresh air – sedentary (FASe, n = 11) 2. Fresh air – interval training (FAIT, n = 11) 3. Cigarette smoke – sedentary (CSSe, n =10) and 4. Cigarette smoke – interval training (CSIT, n = 10). During exposure, the mice were kept in two different chambers (1500U, Tecniplast, Milan, Italy) in groups of 20 and 22 mice and exposed either to cigarette smoke or fresh air respectively. The mice in the CSSe and CSIT groups were given whole body exposure to mainstream smoke from 3R4F research cigarettes (University of Kentucky, Lexington, US) generated by a smoking apparatus previously used by Nilsson et al. [12]. The animals were exposed for 14 weeks, 5 days/week, 6 hours/day. The concentration of the cigarette smoke was monitored (Microdust aerosol monitor AMS950, Casella, Bedford, UK) and the puff intervals in the smoking protocol were adjusted to achieve a concentration of 100–200 mg/m^3^ TPM in the chamber. To monitor levels of carbon monoxide, each time the puff protocol was altered, a mouse was co-exposed with the study animals for six hours before it was anesthetized with isoflurane and an arterial blood sample was drawn from the aorta before the animal was killed. The sample was analyzed (ABL800, Radiometer, Brønshøj, Denmark) and the carboxyhemoglobin (COHb) concentration was within the range 23.1-29.4%. All experiments were performed in accordance with international guidelines and approved by the Norwegian Council for Animal Research.

### Oxygen uptake and exercise

24–72 hours after the end of the exposure period, the animals in the FAIT and CSIT groups were subjected to treadmill interval running for 1 hour/day, 5 days/week for 4 weeks, while the FASe and CSSe groups were kept sedentary. The interval training consisted of uphill running with an inclination of 25° for bouts of 4 minutes at 85-90% of peak work rate alternating with 2 minutes at 50-60% peak work rate on a treadmill customized as previously described [13]. Peak oxygen uptake (VO_2peak_) was measured by treadmill running in a closed metabolic chamber as previously described [13]. This was measured for all animals at the start and the end of the training period. In the CSIT and FAIT groups, the peak oxygen uptake was also measured once per week during the training period.

### Echocardiography

Echocardiography was performed 1–3 days after the last training session (Vevo 770, 35 MHz probe, VisualSonics, Toronto, Canada) with the animals anesthetized with 1.25-2.25% isoflurane, which was adjusted to keep the heart rate and respiration approximately similar between animals. Fractional shortening of the left ventricle (LV) were obtained from M-mode in short axis parasternal view. In B-mode apical four chamber images the M-mode cursor was oriented through the apex and to the lateral tricuspid annulus and the movement of the tricuspid plane, the tricuspid annular plane systolic excursion (TAPSE), was obtained. Isovolumic relaxation time (IVRT) was obtained by examining pulsed Doppler tracing of mitral blood flow in the apical four chamber view. Tissue Doppler early (E’) diastolic velocities, as well as systolic velocity (S’) and RV isovolumic relaxation-time (RV IVRT’) were measured in the intraventricular septum for LV and from the lateral aspect of the tricuspid annulus for the RV. RV wall thickness was obtained from four chamber view. All echocardiographic measurements were averaged over three cardiac cycles. The examiner was blinded for group allocation when reviewing the images from the examination.

### Lung histology and immunochemistry

1–3 days after the echocardiography the mice were anesthetized with isoflurane and killed. The left lobe and the diaphragmatic lobe of the lungs were cannulated with a 21 gauge butterfly needle and fixed with 15 cm H_2_0 pressure of 4% buffered formalin solution for >2 hours. The lung lobes were then kept in formalin solution until they were paraffin embedded, cut and stained with hematoxylin, eosin and saffron. The slides were examined under a light microscope with digital recorder (Olympus BX41/D25, Tokyo, Japan). The lobe with the least artifacts and the most uniform expansion was chosen for further investigation. An enumerated grid with each cell corresponding to a visual field under 200x magnification was applied and 10 fields chosen randomly and photographed for further investigation. A 100 um × 100 um grid was superimposed on the images and the number of transitions from air space to tissue was counted. Areas with vessels or airways >50 um, or with obvious artifacts were excluded, 166(±21) grid lines per animal were counted and the mean value per animal was used in the statistical analysis. Mean linear intercept (MLI), which is an estimate of airspace enlargement, was calculated as described [14]. An increase in MLI corresponds to increasing size of the alveoli as seen in emphysema.

To quantify muscularization of pulmonary vessels sections of lung tissue were stained with antibodies against von Willenbrand factor (vWF) and smooth muscle antigen (SMA), for detailed description see Additional file 1. The sections were then examined (BX40CY light microscope, Olympus) and positive staining with vWF was used to identify pulmonary blood vessels, and staining with SMA was used to assess muscularization of the vessels. Vessels 30–125 μm were scored for SMA-staining, areas adjacent to bronchioles or large hilar vessels were excluded. A score from 1–5 was used to score actin staining: 1 = No actin staining; 2 = Faint actin staining; 3 = distinct staining ≤ ½ of the circumference; 4 = distinct staining ≤ ¾ of the circumference; and 5 = distinct staining of the entire circumference [15]. Per animal 65 ± 21 vessels were scored and the mean score per animal was used in the statistical analysis. All histomorphometric measurements were performed with the examiner blinded to group allocation.

### Statistics

All analyses were done with IBM SPSS Statistics 21. Data is shown as mean ± standard deviation. P-values < .05 were considered significant. Two-way ANOVA was used for all analyses; interaction term was removed if found not to be significant and p-values for main effects were then given. Pre-post exercise VO_2peak_ data were analyzed on delta values (post exercise VO_2peak_ – pre exercise VO_2peak_). Two-way ANCOVA was used to correct for heart rate. For the calculation of MLI outliers were trimmed with Tukey’s outlier filter. Normality was evaluated with Q-Q-plots and Shapiro-Wilk. Equality of variances was evaluated with Levene’s test.

## Results

Body and liver weights are shown in Table 1. There was a significant reduction in body weight due to exercise (p = .007). Compared to the sham exposed animals, liver weight was increased in the smoke exposed, both measured as absolute weight (p = .046) and as weight relative to body weight (p < .001).

**Table 1:** **Body and organ weights after exposure period and exercise period**

	**Fresh air**		**Cigarette smoke**		**Significance**		
	**Sedentary**	**Exercise**	**Sedentary**	**Exercise**	**F1**	**F2**	**I**
Body weight (g)	26.2 ± 1.5	23.4 ± 3.6	24.0 ± 1.8	22.9 ± 1.1	.055	.007	(.21)
Liver weight (g)	.96 ± .06	.97 ± .14	1.02 ± .10	1.02 ± .08	.046	.79	(.95)
Liver weight/body weight (%)	3.6 ± .2	4.1 ± .2	4.3 ± .3	4.5 ± .2	<.001	<.001	(.08)

F1-column: p-value for main effect of smoke exposure; F2-column: p-value for main effect of exercise; I-column: p-values for interaction term between smoke exposure and exercise. 2-way ANOVA, interaction term removed if not significant (p-value for interaction term presented in parenthesis). Data presented as mean ± SD.

### Peak oxygen uptake (VO_2peak_)

**Figure 1:**
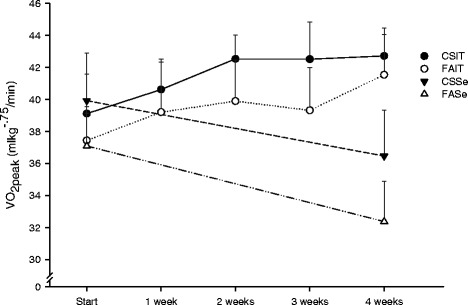
**Peak oxygen consumption (VO**_**2peak**_ **) for mice during treadmill running at the start, during and at the end (4 weeks) of the exercise period.** Both exercise training (p < .001) and smoke exposure (p < .01) increased VO_2peak_. CSIT: cigarette smoke exposed – interval training. FAIT: fresh air exposed – interval training. CSSe: cigarette smoke exposed – sedentary. FASe: fresh air exposed – sedentary. Data presented as mean and SD.

After the exercise period, the exercised animals had higher VO_2peak_ than the sedentary (p < .001). VO_2peak_ was higher in the smoke exposed animals compared to the sham-exposed animals both at start of the exercise period (p = .004), and at the end (p = .002) (Figure 1).

### Emphysema

**Figure 2:**
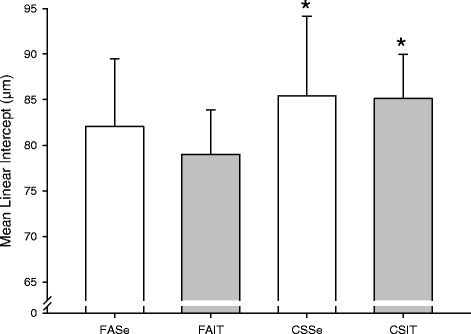
**Alveolar air spaces measured by mean linear intercept in mice exposed to cigarette smoke (CS) or fresh air (FA) and put through 4 weeks of interval training (IT) or kept sedentary for 4 weeks (Se).** Bars indicate mean ± SD. Higher bars represent more severe emphysema. *p = 0.026 (main effect of CS vs FA, 2-way ANOVA without interaction term).

Alveolar air spaces (Figure 2) was significantly increased in mice exposed to cigarette smoke (p = .026); no effect was observed of the exercise intervention (p = .403).

### Echocardiography

Results from echocardiography are shown in Table 2. Smoke exposure significantly reduced systolic function of the right ventricle as shown by a marked reduction of TAPSE (p = .008) and this reduction was attenuated by exercise training (p < .001). Tissue Doppler measurements from right ventricle also showed increase due to exercise both on systolic function RV S’ (p = .004) and diastolic RV E’(p = .007), but here the effect of smoke exposure did not reach significance. There were no significant effects on the measures of left ventricular function. IVRT showed a borderline significant increase due to smoke exposure (p = .074) and a significant decrease due to exercise (p = .004). Tissue Doppler RV IVRT’/RR was borderline significantly increased by smoke exposure (p = .058) and decreased by exercise (p < .001).

**Table 2:** **Assessment of cardiac function by echocardiography**

	**Fresh air**		**Cigarette smoke**		**Significance**		
	**Sedentary**	**Exercise**	**Sedentary**	**Exercise**	**F1**	**F2**	**I**
Heart Rate (stroke/min)	485 ± 30	443 ± 52	444 ± 35	458 ± 33	.27	.26	.024
FS (%)	33.4 ± 3.6	36.5 ± 3.7	35.0 ± 4.0	36.8 ± 6.2	.51	.075	(.63)
LV S’ (mm/s)	21.2 ± 3.4	21.6 ± 3.0	19.6 ± 3.6	19.7 ± 3.8	.11	.84	(.93)
LV E’ (mm/s)	31.3 ± 3.8	33.0 ± 4.3	29.6 ± 6.8	30.2 ± 3.9	.16	.50	(.73)
IVRT (ms)	16.4 ± 1.4	14.1 ± 1.7	16.5 ± 1.6	15.9 ± 1.3	.074	.004	(.09)
TAPSE (mm)	.71 ± .0.04	.82 ± .11	.42 ± .06	.75 ± .12	.008	<.001	(.11)
RV S’ (mm/s)	22.5 ± 5.1	22.0 ± 5.6	20.4 ± 4.9	23.4 ± 3.7	.56	.004	(.078)
RV E’ (mm/s)	31.2 ± 8.3	29.9 ± 6.3	29.15 ± 8.0	31.8 ± 5.1	.51	.007	(.78)
RV IVRT’/RR (%)	8.5 ± 2.3	4.5 ± 1.7	9.5 ± 2.0	6.3 ± 1.6	.058	<.001	(.43)
RV lateral wall thickness (mm)	1.53 ± .40	1.61 ± .34	1.57 ± .35	1.76 ± .31	.50	.33	(.74)

Echocardiography performed on mice exposed to cigarette smoke or fresh air and subsequently put through exercise training or kept sedentary. HR: heart rate; FS: fractional shortening for left ventricle (LV); LV S’: LV Doppler tissue imaging (DTI) systolic velocity; LV E’: LV DTI early diastolic velocity; IVRT: isovolumic relaxation time; TAPSE: tricuspid annular systolic excursion; RV S’: right ventricle (RV) DTI systolic velocity; RV E’: RV DTI early systolic velocity; RV IVRT’/RR: isovolumic relaxation time derived from RV DTI. F1-column: p-value for main effect of smoke exposure; F2-column: p-value for main effect of exercise; I-column: p-values for interaction term between smoke exposure and exercise. 2-way ANOVA, interaction term removed if not significant (p-value for interaction term then presented in parenthesis). Data presented as mean ± SD.

### Muscularization of pulmonary vessels

**Figure 3:**
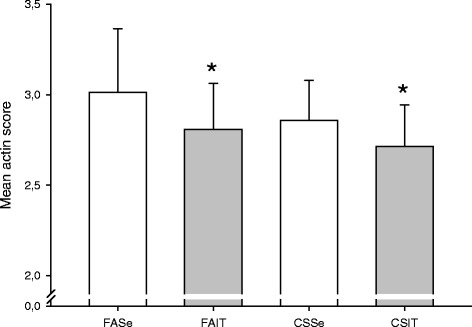
**Muscularization of pulmonary vessels in mice exposed to cigarette smoke (CS) or fresh air (FA) and put through 4 weeks of interval training (IT) or kept sedentary for 4 weeks (Se).** Staining of smooth muscle actin was evaluated on a scale of 1–5 (1 = non-muscularized to 5 = fully muscularized). Bars indicate mean ± SD. *p = 0.040 (main effect of IT vs Se, 2-way ANOVA without interaction-term).

**Figure 4:**
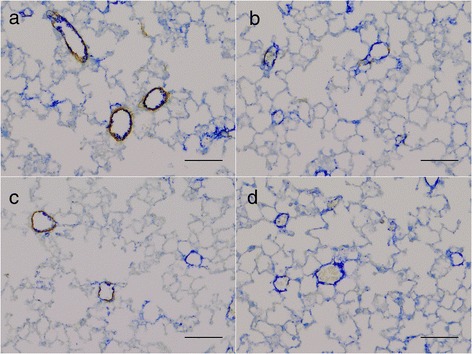
**Sample images showing different degrees of vessel muscularization in lung tissue stained with antibodies against von Willenbrand factor (blue) and smooth muscle actin (brown).** Exercised animals have significantly reduced vessel wall muscularization. **a)** sham exposed, sedentary mice; **b)** sham exposed, exercise trained mice; **c)** cigarette smoke exposed, sedentary mice; **d)** cigarette smoke exposed, exercise trained mice. Scale bars 100 μm.

Smooth muscle in pulmonary vessel walls was reduced by exercise (p = .04), but no significant effect was observed as a result of smoke exposure (p = .14), see Figure 3. Sample images are shown in Figure 4.

## Discussion

In the present study we found that the exposure to cigarette smoke led to hemodynamic dysfunction of the right ventricle in mice and that this dysfunction was reversed after four weeks of aerobic interval training. Muscularization of the pulmonary vessels did not increase from the smoke exposure, indicating that other mechanisms are causing the reduced RV function. We demonstrate that exercise training reduces pulmonary vessel muscularization. Even though the reduced function of the RV was not caused by increased resistance due to increased muscularization of the pulmonary vessels, the reduced muscularization of the pulmonary vessels after exercise training might still contribute to improved RV function through reduced afterload.

### COPD model

We have established a COPD model in mice where we have shown that exposure to cigarette smoke leads to airspace enlargement in the lungs, which indicates emphysematous changes. Models with exposure of mice to smoke generated by burning cigarettes are the most used and characterized animal model of COPD, and female A/J-mice has been shown to develop emphysema with a comparable exposure model [16].

A feature of our model is very high content of carbon monoxide in the inhaled cigarette smoke in exposed animals, leading to high CO-Hb levels. One might argue that the CO-Hb levels are too high to mimic real-life situations, however in order to strain the right ventricle and study the effects of exercise training on the right side of the heart, this model is appropriate.

### Hemodynamics

In this COPD model echocardiography shows a significantly reduced TAPSE, this corresponds to the long-axis shortening of the right ventricle. TAPSE is a widely used and validated marker of right ventricular systolic function in humans [17]. TAPSE has been found to be a robust parameter for right ventricular function in rats, and in a model of pulmonary hypertension it has been shown that reduced TAPSE could be found several days before the development of severe RV failure and could predict the event [18,19]. TAPSE has also been used as a marker of RV function in mice [20,21]. RV IVRT’ was increased by smoke exposure and reduced by exercise training in our study. RV IVRT’ has been shown to correlate well with pulmonary artery pressure in humans, and has been suggested as an alternative to the more conventional echocardiographic assessment of the tricuspid regurgitation for estimating pulmonary artery systolic pressure [22].

The effects of exercise have previously not been studied in animal models or in COPD patients with reduced right ventricular function.

### Pulmonary vessel remodeling

In the present study we have demonstrated that the exercise intervention has led to a decrease in the amount of smooth muscle in the pulmonary vessel walls. Muscularization of pulmonary vessels is a pathological process associated with increased vessel tone and restricted blood flow.

A recent study examined effects on right ventricle and pulmonary vessels in a mouse model with PH induced by 3 weeks of hypoxia. They demonstrated that daily treadmill running during the exposure period prevented an increase in pulmonary vessel muscularization as seen in the untrained animals. They also found that the exercise attenuated the PH induced in the model [23]. The present study reproduces similar effects on pulmonary vessel muscularization, although in another model and with exercise conducted not during but after the exposure period. These results indicate that exercise training not only affect the heart muscle, but also have important effects on the pulmonary vasculature.

We did not find any significant increase in pulmonary vessel muscularization after smoke exposure, but this is previously shown in a similar models with cigarette smoke exposure of mice [15,24,25]. Exposure to CO, in a NO-dependent manner, leads to reduction in pulmonary arterial pressure and reverses pulmonary vessel remodeling in different murine models of pulmonary hypertension [26]. The high CO levels in our model may thus be the explanation why we did not observe increased pulmonary muscularization due to smoke exposure.

We observed a small, non-significant reduction of MLI following exercise. Some previous studies have shown that exercise training may attenuate development of emphysema in smoke exposed mice [27,28]. This suggests that exercise somehow counteracts the destructive effect of smoke exposure on the lung parenchyma and might lead to more preserved alveolar capillaries and a lower total resistance in the pulmonary circulation. This mechanism might reduce afterload and contribute to the exercise induced improvement on RV function observed in this study.

### Peak oxygen uptake

Unexpectedly, we found that smoke exposed animals had higher VO_2peak_ than the sham-exposed. A previous study found that cigarette smoke exposed mice did not perform *worse* than non-exposed mice on treadmill running. It was suggested that this likely was due their model, which mimicked only a mild form of COPD [28]. However, our findings in a somewhat similar model indicate that VO_2peak_ is increased by some unidentified mechanism. A possible explanation for this could be a hypoxia-induced increase of red blood cells in the exposed animals due to high carbon monoxide levels during exposure, which has previously been demonstrated in a rat model of carbon monoxide exposure [29]. The half-life for COHb in mice is 15–20 minutes [30] and the lifespan of the mouse erythrocyte is about 40 days [31]. When the VO_2peak_ testing was performed 28–31 days after the end of the exposure period, COHb would have been eliminated, but increased number of red blood cells from CO-exposure would still be present facilitating increased oxygen carrying capacity.

### Limitations

COPD in humans is defined by spirometric findings, and this is also used for evaluating the severity of the disease. The grading system used for COPD patients is not easily transferable to our model, especially since we did not measure the airway resistance. It is therefore not possible to assess which severity grade of COPD our model will correspond to. However, the induced lung pathology in our model has several commonalities with COPD; exposure to smoke is the most important risk factor for the disease, and emphysema is considered a tell-tale sign of COPD and is perhaps the most important pathological feature of the disease in humans.

In this study most of the data collection was done at a single time-point after the animals had gone through both the exposure and the exercise training. We therefore have little information about how our findings would vary with time and extrapolation of our results to other time-points is uncertain.

Echocardiographic findings give some indices on changes in the pulmonary artery pressure, but this was not measured invasively. Hemodynamic measurements from the right ventricle would have been useful to evaluate the degree of PH.

Isoflurane anesthesia is shown to affect several functional properties of the murine heart [32]. Decreased heart rate is an indicator of depth of anesthesia. The statistical significance level of the echocardiography results did not change after correcting for heart rate (data not shown).

Our findings are made in an animal model and transferability of results to human disease is uncertain and interpretation should be made with care.

## Conclusions

We have demonstrated RV dysfunction in a mouse model of COPD and that this dysfunction was attenuated by interval exercise training. We have further shown reduced muscularization of pulmonary blood vessels after exercise training, which might contribute to improved RV function. It is well documented that exercise training has beneficial effects on heart muscle per se, but the present study suggests that exercise induced alterations in the pulmonary vasculature might contribute to improved cardiac function after exercise intervention in COPD.

## Additional file

Additional file 1:
**Detailed description of immunostaining procedure.**
