# Supplementary material for: Effects of exercise training on pulmonary vessel muscularization and right ventricular function in an animal model of COPD
Source: Respir Res. 2014 Sep 28;15(1):117. doi: 10.1186/s12931-014-0117-y (PMC4181603; doi:10.1186/s12931-014-0117-y)
Supplement: Additional file 1: — Detailed description of immunostaining procedure. [file 12931_2014_117_MOESM1_ESM.pdf]

Additional file 1:

For immunochemistry the sections of lung tissue were dehydrated and then demasked for 20 minutes at 97°C with PT Link/Target Retrival Solution (K8004, Dako, Glostrup, Denmark). Staining was performed with Dako Autostainer Plus (Dako). Sections were first blocked for endogenous alkaline phosphatase and peroxidase with Dual Endogenous Enzyme Block (S2003, Dako) for 5 minutes. The sections were then incubated with SMA (M0851, Dako 1:25) and vWF (A0082, Dako 1:300) simultaneously for 40 minutes, diluted with Antibody Diluent (S2023, Dako). Biotinylated goat-anti-rabbit (E0432, 1:800, Dako) were then applied for 15 minutes before incubation in 1:1 UltraVision Quanto Mouse on Mouse (TL-QHDM, Thermo Fischer Scientific, Waltham, MA, US) and Streptavidin Alkaline Phosphatase (K5005, Dako) for 30 minutes. DAB+ (1:50, K4007 Dako) for 2 x 5 minutes and then Ferangi Blue (1:100, FB813, Biocare Medical, Concorde, CA, US) for 5 minutes were applied for visualization. Sections were rinsed with Wash Buffer (S3006, Dako) between each step. Finally sections were rinsed with water and dried 10 minutes at 60°C and mounted. No counterstaining was applied.
